# Knockdown of LINC01279 Suppresses Gastric Cancer Proliferation and Migration by Inhibiting PI3K/Akt/mTOR Signaling Pathway

**DOI:** 10.1155/2022/6228982

**Published:** 2022-11-08

**Authors:** Weidong Zhao, Xiaohan Zhao, Menglin Xu, Zhengwu Cheng, Zhengxiang Zhang

**Affiliations:** ^1^Department of Oncology, Yijishan Hospital of Wannan Medical College, Wuhu, Anhui, China; ^2^Department of Gastroenterology, Yijishan Hospital of Wannan Medical College, Wuhu, Anhui, China

## Abstract

**Objective:**

To explore the functional and molecular mechanism of long noncoding RNA LINC01279 in gastric cancer (GC).

**Methods:**

The LINC01279 expression in GC and tissues of para-carcinoma was detected by qPCR (real-time fluorescent quantitative PCR), and the association between the LINC01279 expression and clinicopathological features of patients with GC was investigated. The colony formation, CCK-8, transwell assays, and cell cycle detection kit were used for detection of the effect of LINC01279 on GC cell proliferation, cell cycle, colony formation, and invasion. The effect of LINC01279 on PI3K/AKT/mTOR in the GC signaling pathway was identified by the Western blotting technique. The effect of LINC01279 on GC cell proliferation in vivo was evaluated by subcutaneous xenograft tumors in the nude mice.

**Results:**

The results of qPCR displayed the expression of LINC01279 was higher in tissues of GC patients. Furthermore, the tumor size, TNM stage, and metastasis of lymph nodes were also closely related to LINC01279 expression. The experiments on cell function showed that the LINC01279 knockdown significantly inhibited the colony formation, invasion, and proliferation of GC cells and induced the cell cycle arrest in G0 and G1 phases. The Western blotting technique also showed that LINC01279 knockdown significantly inhibited the phosphorylation of PI3K, Akt, and mTOR in GC cells. Furthermore, in vivo experiments displayed that the LINC01279 knockdown significantly inhibited the GC growth.

**Conclusion:**

Knockdown of LINC01279 plays a significant role in inhibiting the PI3K/AKT/mTOR signaling pathway which affects the GC invasion and proliferation. The LINC01279 expression can be utilized as a biomarker for the prediction of the GC prognosis.

## 1. Introduction

The GC is one of the most common cancers with increasing morbidity and mortality. The frequency of gastric cancer is increasing with the increase of the aging population [1, 2]. Due to the occult onset of GC, usually, the patients's diagnosis occurs at later stages, which lead to tumor cell metastasis and seriously affect the efficacy of treatment. The lack of diagnostic and prognostic biomarkers lead to high overall mortality of GC patients, accounting for 14.5% of all cancer-related deaths, which ranked third in cancer-related death [3, 4]. Hence, inspecting the GC pathogenesis and finding new early markers and therapeutic targets that have early diagnostic ability may improve the GC patient's quality of life and their 5-year survival rate.

Non-coding RNA mainly includes long non-coding RNA (lncRNA) and microRNA (miRNA), which exist widely in cells and have physiological functions [5, 6]. Previous findings have revealed that miRNA plays a vital role in gene regulation in various biological and pathological processes. In the past, long noncoding RNA was considered to have had no biological function and was also regarded as the “noise” of the transcription process. However, with the combined results of multiple studies, it was found that lncRNA has tissue and cell specificity and plays a vital role in various body biological processes, including protein interaction, regulation of mRNA shearing, chromatin, and protein remodeling, transcription regulation and others [7, 8]. Studies reported that lncRNA play a significant role in the tumor development or occurrence at many levels, for example, transcriptional, epigenetic, and posttranscriptional regulation. For example, lncRNAs such as lncRNA DANCR, MALAT1, and UCA1 can act as an oncogene to promote tumorigenesis, while other lncRNA such as LOC285194 can inhibit tumorigenesis and tumor development [9–12]. LncRNAs are likely to become biomarkers in the tumor diagnosis, prognosis, and treatment. Other studies have demonstrated that LINC01279 acts as a pericellular substance in endometriosis and can promote the development of endometriosis [13]. However, LINC01279 molecular mechanism and biological function in gastric carcinoma remain unclear. In this study, we elucidate the LINC01279 function and also the related molecular mechanism of LINC01279 in patients GC tissue, in vitro cell model as well as in vivo nude mouse xenograft tumor experiment.

## 2. Materials and Methods

### 2.1. Clinical Sample Collection

This Ethics Committee of Yijishan Hospital of Wannan Medical College approved the present study. All patients signed their consent forms. A collection of 70 pairs of surgically removed para-carcinoma and GC tissues of patients was done who were admitted at the thoracic surgery department of our hospital without any preoperative treatment from February 2018 to February 2020. The PBS buffer was used to wash the tissue samples and stored for 30 min in liquid nitrogen for extraction of RNA from samples. Furthermore, the pathological parameters and clinical information of GC patients are shown in Table 1.

### 2.2. Cell Culture and Transfection

The cell lines of GC including BGC-823, AGS, SGC-7901, MGC-803, MKN-45, and GES-1 (gastric epithelial cells) of normal humans were acquired from the Shanghai Institute of Biological Sciences. Cells were cultured with DMEM or RPMI 1640 medium (Gibco, USA) which contained bovine serum (10%), penicillin (100 U/ml), and streptomycin (100ug/ml) and were incubated at 37°C with a CO_2_ level of 5%. After then, the cells were subcultured at intervals of two days and cultured at 1 : 2~1 : 3 following the cell's growth status. The logarithmic growth phase cells were utilized for investigations and mRNA extraction.

For cell transfection, the negative control (NC) and LINC01279 siRNA were acquired from Sangon Biotech (Shanghai) Co., Ltd. The SGC-7901 cells and MGC-803 cells collection were treated in the logarithmic growth phase, digested with trypsin, resuspended with a complete culture medium, and mixed for making cell suspension. The 6-well plate was used to inoculate the cells (5 × 10^5^/well) for uniform distribution. The fresh basal medium deprived of serum and antibiotics was used to substitute the original medium. The lentivirus transfection solution was prepared according to the grouping, was added to each of the wells, and was mixed thoroughly and placed in the incubator for culturing. After 4~6 h, RPMI-1640 fresh culture medium was used to substitute the transfection mixture, and the plate was again shifted in the incubator for about 1-2 days.

### 2.3. Cell Proliferation Detected by CCK8 Assay

After the cell transfection for 24 h, they were inoculated in 96 well plates (5 × 10^3^/well). The plates were again cultured in incubators for 0, 24, 48, and 72 hours, and then, CCK-8 solution (10 *μ*l) was poured into all the wells, which was placed in 37°C and 5% CO_2_ incubator for 3 h. The absorbance value (OD value) was detected at 490 nm.

### 2.4. Colony Formation Assay

GC cells at 48 h after transfection were cultured in an incubator at 37°C and CO2 level of 5%. After then, they were inoculated into 6-well plates with about 600 cells/well which were added to each well along with 2 ml of culture medium. After every 3^rd^ day, the medium was replaced with the fresh medium. After 2 weeks, PBS buffer was used to wash the colonies and was fixed with paraformaldehyde (4%) at room temperature for 10 min. After then, they were washed three times with PBS buffer, then stained with 1% crystal violet at room temperature for 10 min, then washed three times with PBS buffer. After air drying, the number of colonies (more than 50 cells) was counted under a microscope and repeated three times to calculate the number of colonies in each group.

### 2.5. Analysis of the Cell Cycle

The cell cycle detection kit provided by Beijing Beyotime Biotech Co., Ltd. was used for cell cycle analysis. Briefly, after trypsin digestion and PBS resuspension, 1 × l0^5^ cells in each group were resuspended with 500 *μ*l binding buffer, then 5 *μ*l RNAase, as well as 5 *μ*l propidium iodide, was also added in turn. Afterward, they were incubated in the dark for about 30 min at room temperature. Then, the cell cycle distribution was observed by FC (flow cytometry).

### 2.6. RT-PCR Experiment

According to the collected GC tissue and cell samples, for homogenization, TRIzol reagent (1 ml) was added, and the total RNA was extracted by the kit method. After then, NanoDrop 2000 was used for quantification, and reverse transcription was performed with 200 ng of total extracted RNA through ReverTra Ace qPCR-RT Kit according to the instructions. The qRT-PCR using SYBR ® qPCR Mix was used reverse transcription product analysis. The instrument used in qRT-PCR was CFX96 Touch Real-Time PCR supplied by Bio-Rad. The relative expression level of LINC01279 was calculated by the Ct method. GAPDH was the internal reference gene, and the primer sequences of LINC01279 and GAPDH were referenced [14].

### 2.7. Western Blot

All the samples were added with RIPA lysis buffer, and after homogenization and centrifugation, the total protein extract was obtained, and the protein concentration in the extract was detected by the BCA method. Similarly, the protein electrophoresis was performed through SDS-PAGE, and then, the protein was transferred to the PVDF membrane by 300 mA constant current for 90 min. The PVDF membrane was put into a TBST sealant containing 5% skim milk powder and sealed for 1 h. The primary antibody was incorporated and shaken overnight at 4°C. After washing the membrane, the addition of a secondary antibody before incubation at 37°C for 1 h was done. After washing the membrane, the film was developed.

### 2.8. Subcutaneous Tumor Formation in Nude Mice

Four- to six-week-old BALB/c nude mice, with 18-20 g weight, were obtained from Wannan Medical College (Experimental Animal Center department). The mice were raised in the SPF mouse feeding room with free drinking water and food. All animal feeding and other experimental procedures comply with relevant management and ethical requirements of experimental animals. Every five nude mice were kept in a cage. The animal room conditions were alternating light and shade (12 h illumination/12 h darkness), the relative humidity was kept at (50% ± 10%), the temperature at (22 ± 2) °C, and the facility was regularly disinfected by ultraviolet irradiation. The collection of logarithmic growth phase GC cells was done. Then, the samples were suspended in serum-free medium, adjusted to 1 × 10^6^/ml, and random nude mice were subcutaneously inoculated in the ventral right flank region. The growth and development of nude mice were closely observed. The vernier caliper was used for tumor length and short diameter calculation every day after tumor formation. The growth and development of nude mice were regularly observed, and the growth curve was also plotted. The tumor tissues were stripped and fixed with paraformaldehyde for analysis after nude mice were sacrificed.

### 2.9. Statistical Analysis

The SPSS 19.0 software was utilized to evaluate the collected data. The mean ± standard deviation (*m* ± *s*) was used to express the data. A *T*-test and analysis along with ANOVA were employed for comparing the measurement data with normal distribution, and a nonparametric rank-sum test was used when not obeying normal distribution. The test chi-square was also used for counting data. The value *P* < 0.01 was considered highly significant while *P* < 0.05 was only statistically significant.

## 3. Results

### 3.1. The High LINC01279 Expression in GC Was Correlated with Clinicopathological Features

**Figure 1:**
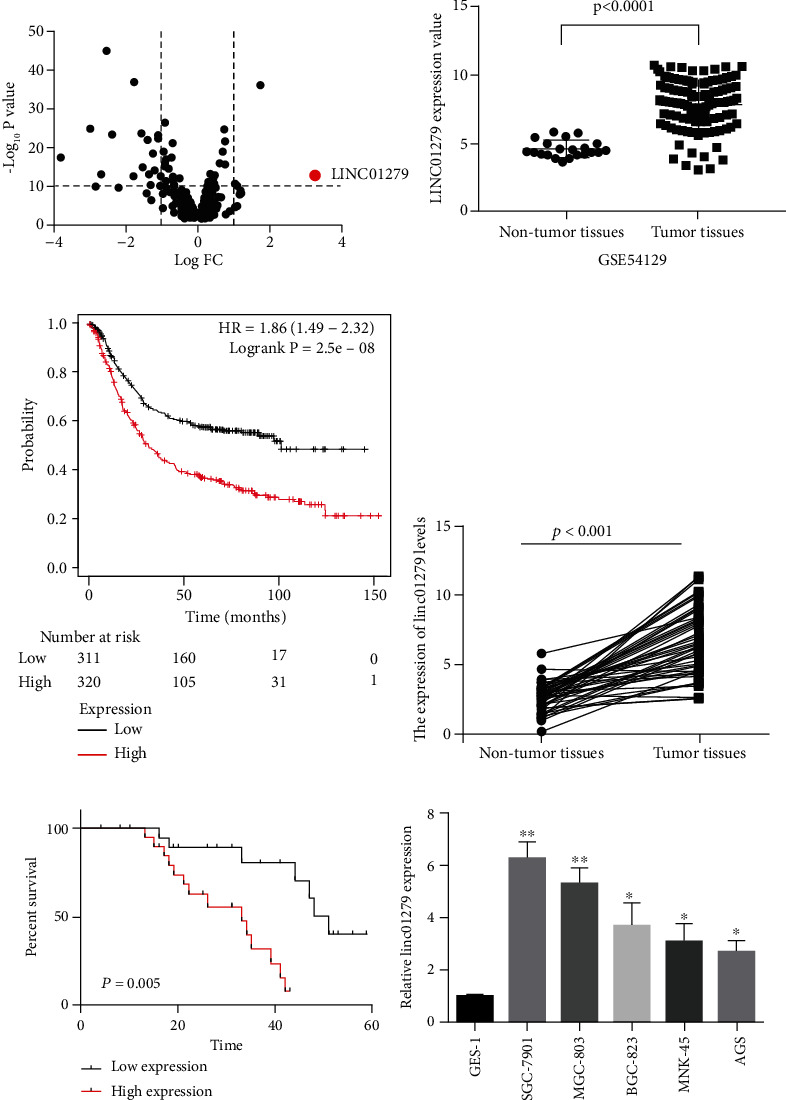
(a) Dataset GSE54129 from the GEO database was utilized to assess the differential expression of noncoding RNA in 111 GC tissues and 21 noncancerous gastric tissues. Fold change values > 2 and −log10 *P* > 10 are used as screening criteria to screen differentially expressed lncRNA; (b) differential expression of LINC01279 in 111 cases of GC tissues and 21 normal gastric tissues (GSE54129 data); (c) Kaplan-Meier plotter database http://kmplot.com/analysis/index.php?p=service&cancer=gastric is used to analyze the expression level effect of LINC01279 on the survival rate of 631 GC patients; (d) fluorescence quantitative PCR detects the LINC01279 expression in 70 cases of GC; (e) analyze the effect of LINC01279 expression on the survival of 70 gastric cancer patients; (f) fluorescence quantitative PCR detects the expression of LINC01279 in GC cell lines (AGS, MKN-45, BGC-823 and SGC-7901) and normal human GES-1. ^∗^*P* < 0.05, ^∗∗^*P* < 0.01.

The noncoding RNA differential expression in the tissue of 111 GC and 21 noncancerous tissues taken from the gastric regions was evaluated using the GSE54129 dataset from the GEO database. Furthermore, the differential expression of lncRNA was screened according to the fold change value > 2 and −log10 *P* > 10. It was found that LINC01279 was significantly overexpressed in GC tissues (red dots, Figures 1(a) and 1(b)). The influence of LINC01279 expression on prognosis in 631 GC patients was analyzed by Kaplan-Meier plotter online database, and it was found that the GC patient's survival rate with more LINC01279 expression was significantly lesser than low expression LINC01279 group (Figure 1(c)). For confirmation of the results, the expression analysis of LINC01279 in 70 GC tissues was done. The results of real-time fluorescent quantitative PCR revealed that the expression of LINC01279 in GC tissues was significantly higher (*P* < 0.05) than in para-carcinoma tissues as shown in Figure 1(d). According to the results of fluorescence quantitative detection, the correlation between LINC01279 expression and patients' clinicopathology was further analyzed. The analysis revealed that the LINC01279 expression was closely related to the TNM stage, tumor size, and lymph node metastasis of GC patients, but age and gender had no significant difference with LINC01279 expression (Table 1). Survival analysis revealed that the GC patients had more LINC01279 expression and were significantly lesser survival rates than the group with low LINC01279 expression in patients as shown in Figure 1(e). This suggests that LINC01279 can be used as an independent prognostic factor. Furthermore, the expression of LINC01279 in GC cell lines including MGC-803, MKN-45, SGC-7901, AGS, and BGC-823 and normal GES-1 was detected by qPCR (fluorescent quantitative PCR). The results demonstrated that the LINC01279 expression level in the cells of GC was significantly more than that in normal GES-1. Furthermore, the highest level of expression was found in MGC-803 and SGC-7901 cells as shown in Figure 1(f). Therefore, the GC cell lines (MGC-803 and SGC-7901) were used for subsequent functional molecular mechanisms and experimental studies. These results indicate that LINC01279 might be associated with GC occurrence and development.

### 3.2. Knockdown of the Expression of LINC01279 Can Play a Vital Role in the Inhibition of GC Cell Proliferation and Colonization

**Figure 2:**
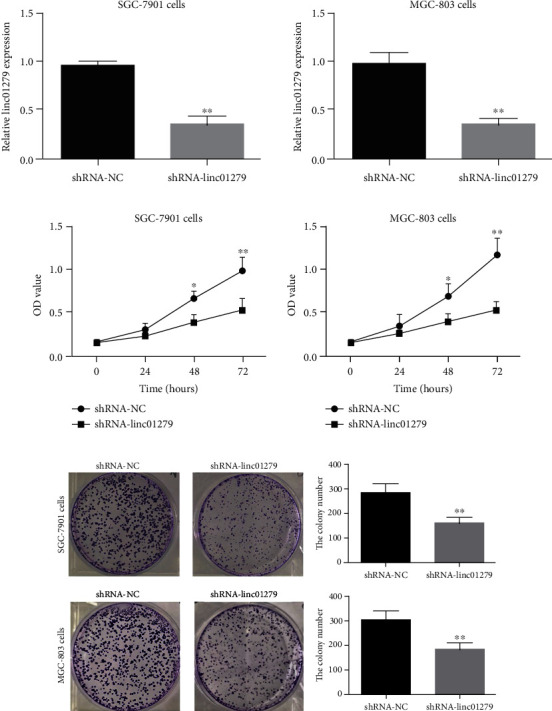
(a) Fluorescence quantitative PCR detects the effect of transfection of shRNA targeting LINC01279 on the expression level of LINC01279 in GC cells SGC-7901 and MGC-803; (b) the detection of LINC01279 knockdown effect on the proliferation of GC cells SGC-7901 and MGC-803 by CCK-8; (c) the colony formation experiment detects the effect of LINC01279 knockdown on the clonogenic ability of gastric cancer SGC-7901 and MGC-803 cells. ^∗^*P* < 0.05, ^∗∗^*P* < 0.01.

Firstly, an interfering shRNA that knocks down LINC01279 was constructed. After transfection of MGC-803 cells and SGC-7901 cells, LINC01279 knockdown efficiency in cells was evaluated, and the specific functions of LINC01279 in GC cells were examined. The result revealed that the transfection of shRNA targeting LINC01279 (shRNA-LINC01279) significantly downregulated the LINC01279 expression level in GC cells than the control group (negative control, shRNA-NC) as shown in Figure 2(a). CCK-8 assay was used for detection of the LINC01279 knockdown effect on the MGC-803 and SGC-7901 proliferation of GC cells. The result revealed that the knockdown of LINC01279 (shRNA-LINC01279) played a significant role in the inhibition of gastric cancer cell proliferation as compare to the control group (shRNA-NC) as shown in Figure 2(b). In the colony formation assay, knockdown of LINC01279 (shRNA-LINC01279) plays a significant role in the inhibition of GC cell's clonogenic ability as compare to the control group (shRNA-NC) as shown in Figure 2(c). The above also revealed that LINC01279 knockdown also played a vital role in the inhibition of GC clonogenic ability and proliferation.

### 3.3. Knockdown of LINC01279 Induced the Cell Cycle Arrest at G0/G1 Phase in GC Cells

**Figure 3:**
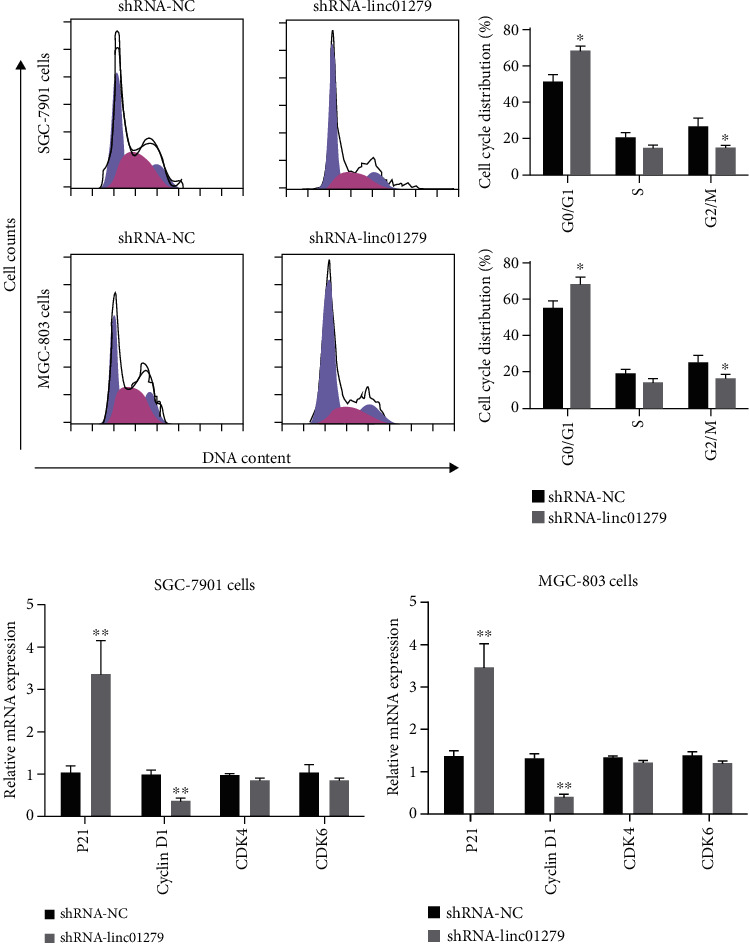
Knockdown of LINC01279 induced the cell cycle arrest at G0/G1 phase in GC cells. (a) Flow cytometric analysis detected cell cycle distribution of LINC01279 knockdown on gastric cancer SGC-7901 and MGC-803 cells; (b, c) fluorescence quantitative PCR was used to detect the expression of G0/G1 phase-related regulatory proteins, such as p21, cyclin D1, CDK4, and CDK6. ^∗^*P* < 0.05, ^∗∗^*P* < 0.01.

Flow cytometry was utilized for the detection of the cell cycle distribution of LINC01279 knockdown on GC MGC-803 and SGC-7901 cells. The result displayed that in comparison with the control group (shRNA-NC), the knockdown of LINC01279 can significantly induce cycle arrest of gastric cancer cells in the G0/G1 phase. Specifically, in SGC-7901 cells, G0/G1 increased from 55.6% in the shRNA-NC group to 71.8% in the shRNA-LINC01279 group. The corresponding S phase and G2/M decrease accordingly. In MGC-803cells, G0/G1 increased from 58.4% in the shRNA-NC group to 69.3% in the shRNA-LINC01279 group (Figure 3(a)). In addition, fluorescence quantitative PCR was used to detected the expression of G0/G1 phase-related regulatory proteins, such as p21, cyclin D1, CDK4, and CDK6. The results showed that compared with the blank control group (shRNA-NC), knockdown of LINC01279 can significantly increase the expression of p21 and inhibit the expression of cell cycle-related protein Cyclin D1, but has no significant effect on the expression of other proteins such as CDK4 and CDK6 (Figures 3(b) and 3(c)). The results as mentioned above indicate that LINC01279 knockdown shows significant results in the inhibition of cell cycle arrest of gastric cancer cells in the G0/G1 phase.

### 3.4. Knockdown of LINC01279 Inhibited the Invasion and Migration of Gastric Cancer Cells

**Figure 4:**
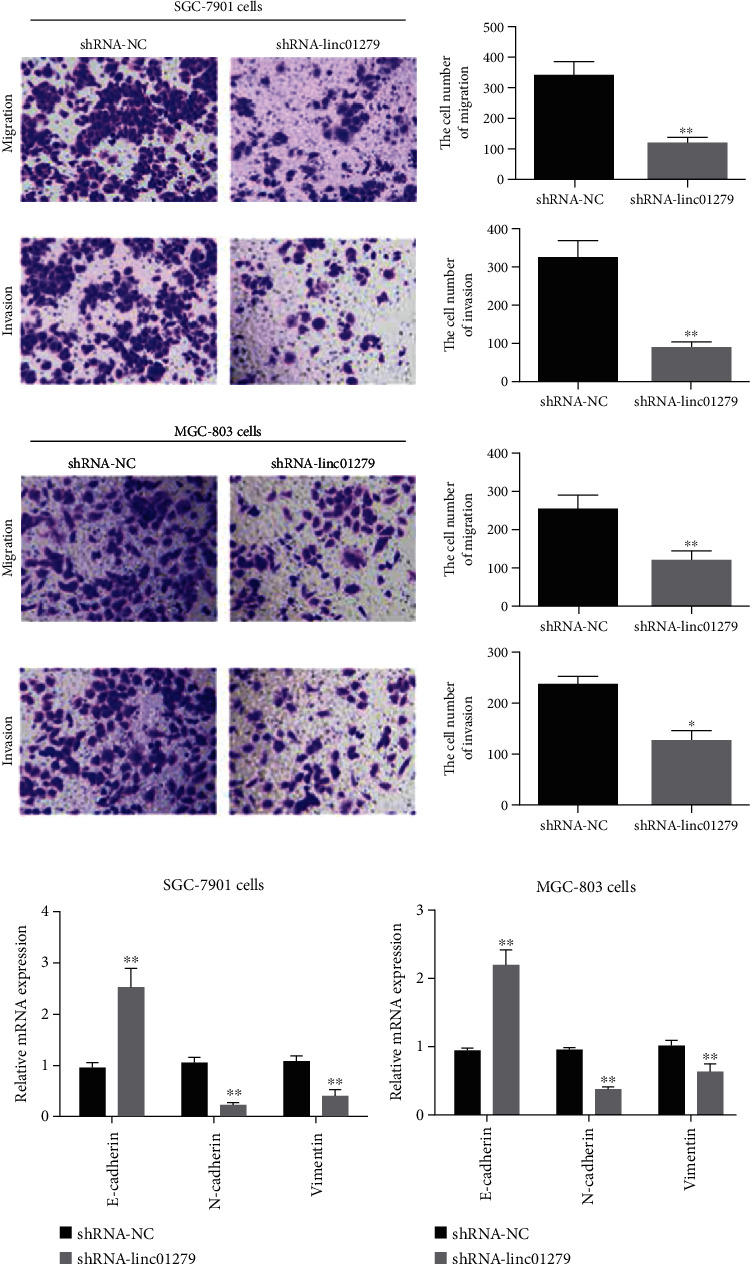
Knockdown of LINC01279 inhibited the invasion and migration of gastric cancer cells. (a) Transwell assay detected the LINC01279 knockdown effect on the invasion and migration of gastric cancer SGC-7901 and MGC-803 cells; (b, c) Fluorescence quantitative PCR was used to detected the expression of key proteins in the process of cell invasion and migration. ^∗^*P* < 0.05, ^∗∗^*P* < 0.01.

The main causes of death in cancer patients are invasion and migration. Therefore, the Transwell assay is used to further detect the effect of LINC01279 on the invasion and migration of gastric cancer MGC-803 and SGC-7901 cells. The results showed that LINC01279 (shRNA-LINC01279) knockdown plays a significant role in the inhibition of GC SGC-7901 and MGC-803 cells, invasion, and migration in comparison with the control group (shRNA-NC) (Figure 4(a)). In order to further evaluate whether knockdown of LINC01279 affects the changes of key proteins in the process of cell invasion and migration ability, the results showed that, compared with the blank control (shRNA-NC), knockdown of LINC01279 can significantly promote the expression of E-cadherin, and inhibited the expression of N-cadherin and vimentin (Figures 4(b) and 4(c)).

### 3.5. Knockdown of LINC01279 Significantly Inhibited the PI3K/AKT/mTOR Signaling Pathway

**Figure 5:**
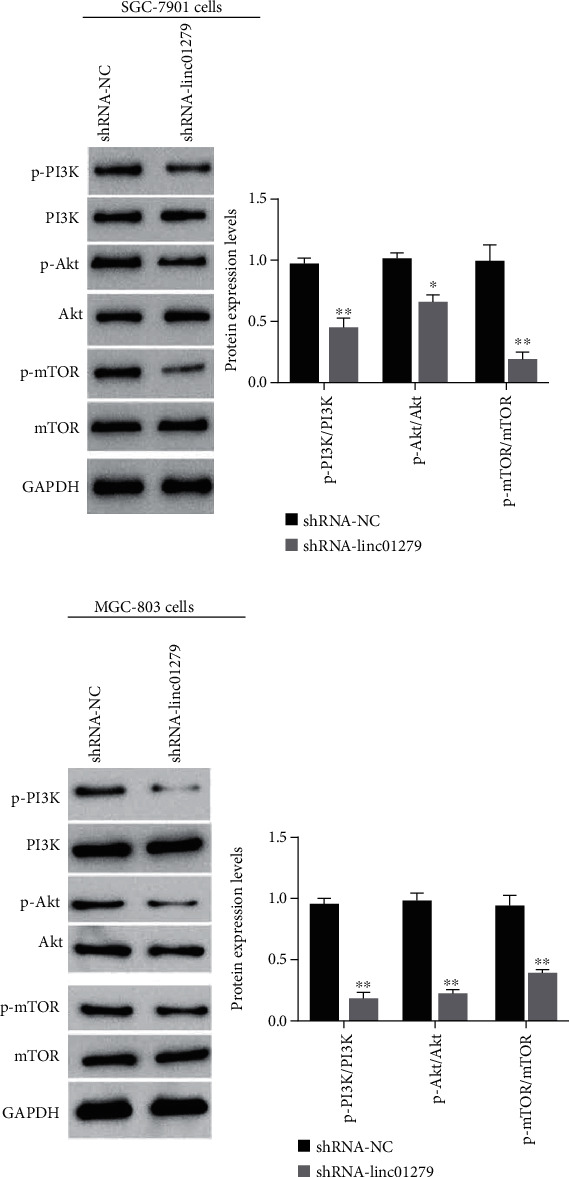
Western blot detected the effect of LINC01279 knockdown on the PI3K/AKT/mTOR in GC SGC-7901 (a) and MGC-803 cells (b), ^∗^*P* < 0.05, ^∗∗^*P* < 0.01.

The signaling pathway “PI3K/AKT/mTOR” also plays a dynamic role in the regulation of GC occurrence and development. The Western blotting analysis presented that knockdown of LINC01279 (shRNA-LINC01279) significantly inhibited the expression levels of p-PI3K, p-Akt, and p-mTOR compared with the blank control group (shRNA-NC) as shown in Figures 5(a) and 5(b). The above results suggested that knockdown of LINC01279 can significantly inhibit the PI3K/AKT/mTOR in GC cells.

### 3.6. LINC01279 Knockdown Significantly Inhibited the GC Growth in Nude Mouse

**Figure 6:**
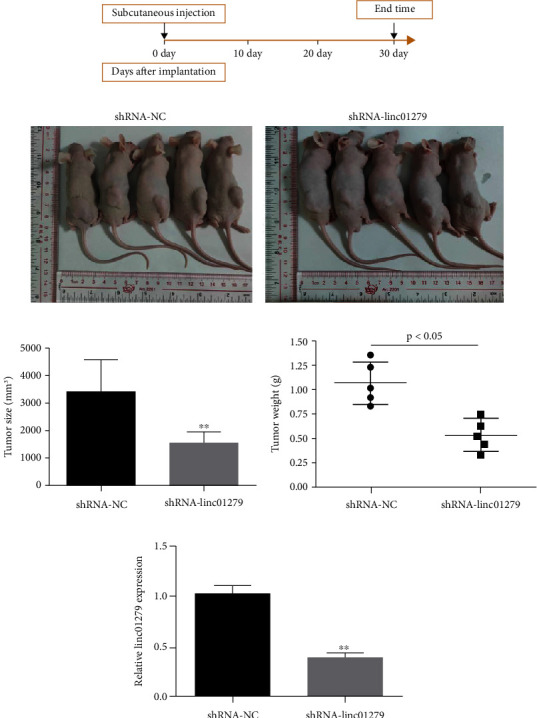
The effect of LINC01279 knockdown on the growth of GC cells (SGC-7901) in nude mice. (a) The timeline of subcutaneous tumor formation in nude mice; (b) the tumors formed under the nude mice's skin, 30 d after inoculation of SGC-7901 cells with different treatments; (c) the tumors were measured and analyzed; (d) after scarification, the tissue of the tumor was stripped and weighed, and the weight of the tumors were analyzed; (e) fluorescence qPCR analyzes the LINC01279 expression level of in tumors. ^∗∗^*P* < 0.01, ^∗^*P* < 0.05.

To further explore the LINC01279 knockdown effect on the growth of GC in vivo, a xenograft tumor mouse model was established (Figures 6(a) and 6(b)). The results were shown in Figure 6(c): knockdown of LINC01279 played a significant role in the inhibition of SGC-7901 cell growth in mice. The volume of tumor formed by SGC-7901 cells in the blank control group (shRNA-NC) was (3451.4 ± 1293.9 mm^3^), and the volume in the knockdown shRNA-LINC01279 group was (1591.7 ± 653.6 mm^3^). Statistical analysis of the data revealed that LINC01279 knockdown significantly inhibited the proliferation of GC cells in nude mice. The tumor tissue was weighed, and the results revealed that in comparison with the control group (shRNA-NC), the knockdown of LINC01279 shows a significant reduction in tumor weight (Figure 6(d)). The fluorescence qPCR analysis revealed that the LINC01279 expression level in tumor tissues was significantly decreased in the shRNA-LINC01279 group in comparison with the control group (shRNA-NC) (such as 6E). Our findings indicated that the LINC01279 knockdown significantly inhibited the growth and development of GC in the mice.

## 4. Discussion

A large number of abnormally expressed lncRNAs can either inhibit or promote the occurrence as well as the development of GC. The mechanism of some lncRNAs are revealed in recent studies and these lncRNAs are anticipated to be biomarkers for early GC diagnosis or therapeutic targets [15–17]. For example, lncRNA CADM1-AS1 has low expression in gastric cancer tissue, and patients with higher expression have better overall survival (OS) and progression-free survival (PFS). Univariate and multivariate analyses showed that lncRNA CADM1-AS1 expression is an independent prognostic indicator for GC patients [17]. In addition, lncRNAs are also involved in regulating tumor angiogenesis, apoptosis, invasion, and drug resistance [18–20]. In this study, we found that the LINC01279 expression is abundant in GC tissue and cell lines. The LINC01279 knockdown play a significant role in the inhibition of gastric cancer cell proliferation and colony formation and induce cell cycle arrest in G0/G1 phase. In vivo xenograft, tumor mouse model demonstrated that the LINC01279 knockdown inhibits the GC growth.

LINC01279 is a newly identified regulatory molecule, whose abnormal expression is correlated with the occurrence of endometriosis and strabismus [13, 21]. LINC01279 can promote the development of endometriosis [13]. The results of this study show that LINC01279 is upregulated significantly in both GC tissue and cells. The expression of LINC01279 is closely related to the tumor size, TNM stage, and lymph node metastasis in gastric cancer patients. Meanwhile, LINC01279 can be used as an independent prognostic factor.

It has been found that lncRNAs play a vital role in the regulation of tumor development at multiple levels. LncRNA MEG3 is expressed at a low level in gastric cancer tissues, and overexpression of this gene inhibit the metastasis and proliferation of GC cells by increasing the expression of p53 [20]. lncRNA HOXA11-AS can inhibit the ability of EZH2 protein translation through the action of the miRNA-1297 sponge. At the same time, lncRNA HOXA11-AS can also regulate the chromatin modifiers PRC2, LSD1, and DNMT1 to promote GC invasion and proliferation [22]. It is shown in this study that LINC01279 knockdown plays a significant role in inhibiting the proliferation and clonogenic ability and induces cell cycle arrest of gastric cancer cells in the G0/G1 phase.

The PI3K/AKT/mTOR signaling pathway is frequently activated and considered as a promising therapeutic target in many tumors, including GC. Inhibition of this signaling pathway can significantly inhibit tumor proliferation and invasion [23, 24]. For example, the dual inhibitor BEZ235 overcomes the GC paclitaxel (PTX) resistance by targeting the PI3K/Akt/mTOR signaling pathway [25]. Other genes can also regulate the GC occurrence and development by activating or inhibiting the PI3K/Akt/mTOR signaling pathway. The NUCKS1 activates the PI3K/Akt/mTOR through upregulation of IGF-1R expression, thereby promoting gastric carcinogenesis [26]. lncRNA XLOC_006753 promotes the occurrence of GC multidrug resistance by activating PI3K/Akt/mTOR pathway [27]. In this study, it is found for the first time that knockdown of LINC01279 can significantly inhibit the PI3K/Akt/mTOR signaling pathway. In vivo tests in nude mice show that LINC01279 knockdown can significantly inhibit GC growth in vivo. The molecular mechanism of how LINC01279 regulates the PI3K/Akt/mTOR pathway is currently unclear, which warrants further study.

In conclusion, the expression of LINC01279 is significantly higher in GC tissue and cell lines. LINC01279 knockdown has significant effects on the inhibition of gastric cancer cell proliferation and colony formation and induces cell cycle arrest in the G0/G1 phase. Furthermore, in vivo tests show that LINC01279 knockdown inhibits the growth of GC in nude mice. Therefore, LINC01279 could be a new target for the future treatment of GC.

## Figures and Tables

**Table 1:** The correlation between clinical characteristics of GC patients and LINC01279 expression.

Characteristics	Cases (*n* = 70)	LINC01279 expression		*P* value
		Low	High	
Gender				
Male	37	16	21	0.8015
Female	33	16	17	
Age				
>60 years	32	17	15	0.6316
≤60 years	38	17	21	
Tumor size				
≤3 cm	43	25	18	0.0060^∗^
>3 cm	27	6	21	
Histological grade				
High, middle	29	16	13	0.6469
Low	41	25	26	
Local invasion				
T1-T2	34	20	14	0.0929
T3-T4	36	13	23	
Lymph node metastasis				
Negative	25	19	6	0.0004^∗^
Positive	45	14	31	
TNM stage				
I-II	33	20	13	0.0299^∗^
III-IV	37	12	25	

^∗^ indicates statistically significant.

## Data Availability

Emails could be sent to the address below to obtain the shared data: zhaowd@wnmc.edu.cn.
